# Polysaccharide-K augments docetaxel-induced tumor suppression and antitumor immune response in an immunocompetent murine model of human prostate cancer

**DOI:** 10.3892/ijo.2011.1292

**Published:** 2011-12-12

**Authors:** CYNTHIA A. WENNER, MARK R. MARTZEN, HAILING LU, MICHAEL R. VERNERIS, HONGBO WANG, JOEL W. SLATON

**Affiliations:** 1 Bastyr University Research Institute, Kenmore, WA 98028; 2 Departments of Pediatric Blood and Marrow Transplantation and Urologic Surgery, University of Minnesota, MN 55455; 3 Tumor Vaccine Group, Center for Translational Medicine in Women's Health, University of Washington, Seattle, WA 98109, USA

**Keywords:** prostate cancer, polysaccharide-K, docetaxel, apoptosis, natural killer cell

## Abstract

Advanced castration-resistant prostate cancer has high mortality rates and limited treatment options. Novel therapies are needed to better contend with this disease. Polysaccharide-K^®^ (PSK), an extract of the mushroom *Trametes versicolor*, has immunomodulatory and tumor suppressive activities. PSK is used in Asia as a cancer immunotherapy. However, its benefit in combination with taxanes for prostate cancer is unknown. We examined whether PSK would enhance docetaxel-induced apoptosis and augment anti-tumor immune responses in orthotopic tumors using transgenic adenocarcinoma of the mouse prostate (TRAMP)-C2-bearing mice. Combining PSK with docetaxel induced significantly higher tumor suppression than either treatment alone (p<0.05), including a reduction in tumor proliferation and enhanced apoptosis. Combined PSK and docetaxel treatment led to a lower decrease in number of white blood cells than docetaxel alone, an effect accompanied by increased numbers of tumor-infiltrating CD4^+^ and CD8^+^ T cells. PSK with or without docetaxel significantly enhanced mRNA expression of IFN-γ compared to control, but did not significantly alter T-regulatory FoxP3 mRNA expression in tumors. PSK also augmented docetaxel-induced splenic natural killer cell cytolytic activity against YAC-1 target cells (p=0.045). This study is the first to show that PSK enhances docetaxel-induced prostate cancer tumor suppression, apoptosis and antitumor responses.

## Introduction

Prostate cancer is the second most common cancer with the fifth highest age-standardized mortality rate in men worldwide (1). Unlike localized disease, which is curable via a number of therapeutic strategies, advanced prostate cancer that has metastasized and is hormone-resistant has limited therapeutic options that offer only temporary disease control (2).

In 2005, the FDA approved docetaxel for the treatment of metastatic castration-resistant prostate cancer after two randomized clinical trials demonstrated improved survival over then standard-of-care mitoxantrone and prednisone therapy (3,4). Docetaxel chemotherapy has become standard treatment and the building block for novel therapeutic approaches to metastatic, hormone-refractory disease. Docetaxel acts by stabilizing microtubules in mitotic spindles, directly inhibiting the cell division process (5,6). Because docetaxel is cytotoxic to all dividing cells, it induces myelosuppression and neutropenia at therapeutic doses (7,8), limiting its therapeutic potential.

Immunotherapies offer a potential means to enhance the clinical efficacy of docetaxel by inducing antitumor immune responses in conjunction with docetaxel's direct antitumor effects. Several immunotherapies are currently under development or in use in cancer treatment. The dendritic cell vaccine sipuleucel-T (Provenge^®^) was approved by the FDA in 2010 after a phase III trial showed a 38% increase in 3-year overall survival with a median survival advantage of 4.1 months compared to placebo in men with metastatic castration-resistant prostate cancer (CRPC) (9). Clinical trials combining vaccine therapy and docetaxel are in progress (10). A second even more recently FDA-approved immunotherapeutic drug, ipilimumab (Yervoy^®^), a humanized anti-CTLA-4 drug, showed improved survival in melanoma patients and several trials to assess its efficacy in prostate cancer have been done or are underway (11–15). These promising immunotherapies support the hypothesis that combining pharmacological agents with novel immune therapies can lead to tumor suppression and survival benefit.

Polysaccharide-K (PSK) is a pharmaceutical grade extract of the mushroom *Trametes versicolor* used throughout Asia as an adjunctive cancer treatment due to its reported direct antitumor effects, antimetastatic actions and immune modulatory properties (16). PSK has recently been shown to have TLR2-agonist activities and to significantly inhibit breast cancer growth in neu transgenic mice in a CD8^+^ T cell- and NK cell-dependent manner (17). A role of NK cells in the antitumor actions of PSK is further supported by results from an earlier study showing that PSK lengthened tumor doubling times and increased mean survival times in T cell-deficient, but not NK-deficient mice bearing human prostate tumors (18). A number of randomized prospective clinical trials testing oral administration of PSK combined with chemotherapy demonstrated improved survival for lung (19,20), breast (21), gastric (22,23), and colorectal (24) cancers. PSK in combination with docetaxel has shown enhanced antitumor activity against human pancreatic (25) and gastric (26) cancer cells, and in murine xenograft models of gastric cancer (26,27). These findings together suggest that combining PSK with docetaxel chemotherapy may enhance docetaxel's tumor suppressive actions and further suggest that PSK addition may increase T cell and NK cell-dependent antitumor responses.

Despite these promising preliminary data, the effects of oral administration of PSK on docetaxel-induced antitumor responses in an immunocompetent prostate cancer setting are as yet unknown. This study was conducted to investigate whether combining orally administered PSK with docetaxel is safe and whether it enhances docetaxel-induced therapeutic responses and antitumor responses in TRAMP-C2 prostate tumor-bearing mice.

## Materials and methods

### Reagents

PSK was donated by Kureha Chemical Industry (Tokyo, Japan) and docetaxel obtained from Sanofi-Aventis USA (Bridgewater, NJ).

### Antibodies and staining reagents

CD4 and CD8 primary Abs were obtained from Santa Cruz Biotechnology (Santa Cruz, CA). Anti-Ki67 was obtained from Dako (Copenhagen, Denmark). The TUNEL staining kit was obtained from R&D Systems (Minneapolis, MN). The ABC Vector staining kit (Vector Labs, Burlingame, CA) was used for fluorescent secondary staining in some cases. Rabbit polyclonal IgG against cleaved caspase-3 (Asp 175) (specific for mouse, rat and human cleaved caspase-3) was obtained from Cell Signaling Technology (Beverley, MA).

### Murine prostate cancer model and therapy

Mouse prostate tumor (TRAMP-C2) cells (American Type Culture Collection, Manassas, VA) were injected orthotopically into C57BL/6 mice (Charles River, Wilmington, MA) following a study protocol approved by the University of Minnesota Institutional Animal Care and Use Committee. This procedure was performed under general anesthesia (isoflurane). Tumors were allowed to grow for approximately two weeks. Tumor growth was monitored by palpation of the prostate; established tumors present as hardened masses at the injection site. After this period, mice were randomly assigned to one of four treatment groups: 1) saline control; 2) docetaxel only; 3) PSK only; 4) docetaxel plus PSK. Control mice received saline daily through oral gavage, as well as twice weekly saline intraperitoneal (i.p.) injections. The docetaxel only treatment group received docetaxel (5 mg/kg) injected i.p. twice weekly and saline vehicle control daily by oral gavage. The PSK only treatment group received PSK (300 mg/kg) daily by oral gavage and i.p. injections of saline twice weekly. The docetaxel + PSK treatment group received PSK (300 mg/kg) daily by oral gavage and docetaxel (5 mg/kg) i.p. injections twice weekly. Injections were administered in 100 μl volumes. Mice were weighed every other day. Groups were treated for 11–13 days before mice were sacrificed and specific organs harvested for end-point monitoring. Prostate tumors were excised and weighed, and tumors and spleens were used to determine concentration and activity of different immune cell types. Excised tumors were also analyzed for proliferation and apoptosis. Blood was also collected to determine white blood cell counts and assay hepatic and renal function for safety assessment.

### Hematoxylin and eosin staining of tissue

To confirm the presence of tumor, frozen sections of tumor tissue (4 micron) were subjected to hematoxylin and eosin staining. Sections were placed into distilled water and nuclei stained with alum hematoxylin for 4 minutes (min), then rinsed with water. Sections were then treated with 0.3% acid alcohol and rinsed. Slides were then treated with eosin for 2 min, rinsed, dehydrated, mounted and examined for tumor presence.

### Immunohistochemistry for Ki67 and TUNEL apoptosis assay

Unstained serial frozen sections of tumor were cut to 4-micron thickness and placed into phosphate buffered saline (PBS). Sections were exposed to 1–1,000 dilution of Ki67 (Dako) and incubated for 1 hour (h) at 37°C. All slides were incubated with the appropriate biotinylated secondary antibody: goat anti-rabbit IgG (Vector), for 20 min at 37°C. The cells were then counted as described below. Other nearby sections were used for immuno-fluorescent staining to detect the presence of apoptosis using the TUNEL apoptosis assay kit (R&D Systems) according to the manufacturer's protocol. Briefly, slides were treated with proteinase K for 30 min at 37°C and incubated with a terminal deoxynucleotidyl transferase end labeling cocktail (terminal deoxynucleotidyl transferase buffer, biotin-dUTP, and terminal deoxynucleotidyl transferase at a ratio of 90:5:5) for 120 min at 37°C. This was followed by overlaying an avidin-FITC (green) solution (50 μl) and incubating in the dark for 60 min at 37°C.

### Determination of WBC counts

At the time of sacrifice, mice were sedated and heart punctured with 25 gauge needle and 500–1,000 μl blood removed for WBC analysis using a Coulter Counter (Beckman Coulter, Brea, CA).

### Liver and renal function analysis

To assess toxicity of the different treatments, plasma samples obtained from mice (N=4) in each treatment group at time of sacrifice (11–13 days post-treatment) were tested for standard parameters of liver and renal function, including alanine transaminase (ALT), aspartate aminotransferase (AST), creatinine (Cr), blood urea nitrogen (BUN), albumin and total protein levels.

### Cytokine and cytolytic gene and Foxp3 mRNA expression

To assess expression of IFN-γ, IL-2, TNF-α, TGF-β, perforin, granzyme B and FoxP3 genes, ribonucleic acid (RNA) was isolated from tumors using the RNAqueous4PCR kit (Applied Biosystems/Ambion, Austin, TX). The integrity of RNA was tested using an Agilent Bioanalyzer (Agilent Technologies, Palo Alto, CA). Complementary deoxyribonucleic acid (cDNA) was generated from 5 μg of RNA using Superscript III reverse transcriptase (Invitrogen, San Diego, CA) with oligo-dT as primers according to the manufacturer's protocol. The cDNA (5 μl) diluted 1:40 was used as the template for real-time PCR analysis. The primers and probes (FAM-MGB) for TaqMan-based gene expression assays were purchased from Applied Biosystems (Foster City, CA). Real-time PCR was performed in 384 well thin-wall PCR plates using ABI PRISM 7900HT under the following conditions: initial denaturation at 95°C for 10 min, followed by 40 cycles of denaturation at 95°C for 15 sec and a combined annealing/extension step at 60°C for 1 min. Data analysis was performed using SDS 2.21 (Applied Biosystems). The messenger RNA (mRNA) expression level of the target gene was normalized to β-actin using the ΔC_T_ method. Level of expression=2^−ΔCT^, where ΔC_T_=C_T target gene_−C_T actin_. CT is the cycle threshold at which the fluorescence signal crosses an arbitrary value.

### Flow cytometry for immunophenotyping and apoptosis analysis of spleen cells

After sacrifice, spleens were removed and each spleen rendered to a single cell suspension using standard techniques (28). Red blood cells were lysed with hypotonic media (ACK lysing buffer, Lonza, Inc. Walkersville, MD) for 3–5 min and then mononuclear cells were washed with PBS two times. Approximately 10^5^–10^6^ cells were stained on ice with Fc block (BD Pharmingen, San Diego, CA) for 15 min, followed by addition of fluorochrome-labeled mAbs to specified cell surface markers for immunophenotyping, and annexin V and propidium iodide for apoptosis analysis. Cells were washed and resuspended and analyzed by a FACSCanto flow cytometer and FlowJo software (BD Biosciences, San Jose, CA).

### Dual fluorescence immunohistochemistry for caspase-3 and CD4/CD8

Sections of frozen tumors (6–8 from each group) were cut to 4 micron thickness. Immediately prior to immunolabeling, sections were re-fixed in cold acetone, equilibrated in PBS-saponin and incubated with 5% normal goat (or donkey) serum for 1 h at 37°C. After washing, they were incubated with rabbit anti-caspase-3 (1:75) for 48–60 h at 4°C, followed by addition of goat anti-rabbit IgG biotin (1:100; Jackson ImmunoResearch Laboratories, West Grove, PA) for 1 h at 37°C. The anti-caspase-3 Ab detects only the large fragment of activated caspase-3 (17–19 kDa) that results from cleavage after Asp 175 and does not recognize other caspases. Sections were routinely washed in excess PBS, pH 7.5, containing 0.3% saponin (Sigma-Aldrich, St. Louis, MO) at the end of each incubation step, and the same buffer was used as a diluent in all immunohistochemical reagents for caspase-3. These sections were incubated with streptavidin Texas red (1:100; Jackson ImmunoResearch Laboratories) for 1 h at 37°C. Each section was then incubated with rat antibodies to either CD4 or CD8. Sections were then washed with PBS and exposed to goat anti-rat secondary antibodies and pig IgG-FITC (1:50; Jackson ImmunoResearch Laboratories). Sections were then washed and prepared for fluorescence microscopy. Slides were examined with a Nikon TE2,000-U fluorescent microscope (Nikon Corp., Tokyo, Japan). Images were digitized by Photoshop 8.0. Ten high powered fields (×100) from 3–4 slides from each of 6–8 tumors per treatment group were viewed for the presence of fluorescently labeled cells: red (cleaved caspase-3), green (CD4 or CD8 in 2 separate slide sets) or dual-expressing yellow. Numbers of positive cells were counted and mean cell number ± standard error of the mean reported. The individual counting the cells was blinded to the treatment group from which sections were taken.

### NK cytolytic activity

At the time of sacrifice, splenocytes were isolated and assessed for NK cell cytolytic activity against YAC-1 tumor cells by a standard flow cytometric assay. YAC-1 cells labeled with 3,3′-dioctadecyloxacarbocyanine [DiOC_18_(3)] purchased from Sigma were cultured with splenocytes for 4 h in triplicate at the effector to target cell (E:T) ratios of 50:1, 25:1, 12.5:1 and 6.25:1. After 4 h, cells were stained with propidium iodide (PI) and washed prior to flow cytometric analysis. Dead target cells were detected as DiOC^+^PI^+^ cells. NK cell activity, defined as percent specific cytotoxicity, was calculated at each E:T ratio using the following formula: (% DiOC^+^, PI^+^ cells) _Splenocytes+YAC-1_ - (% DiOC^+^, PI^+^ cells) _YAC-1 alone_ for each replicate sample. Data were analyzed for significant differences using a linear mixed model to analyze data from 17 mice.

### Statistical analysis

Continuous variables, unless otherwise noted, were analyzed using the Student's t-test. Non-continuous variables (tumor weights) were analyzed using the Mann-Whitney U test. Statistical significance was set at p≤0.05.

## Results

### PSK enhances docetaxel-induced tumor suppression and apoptosis of tumor cells

**Figure 1:**
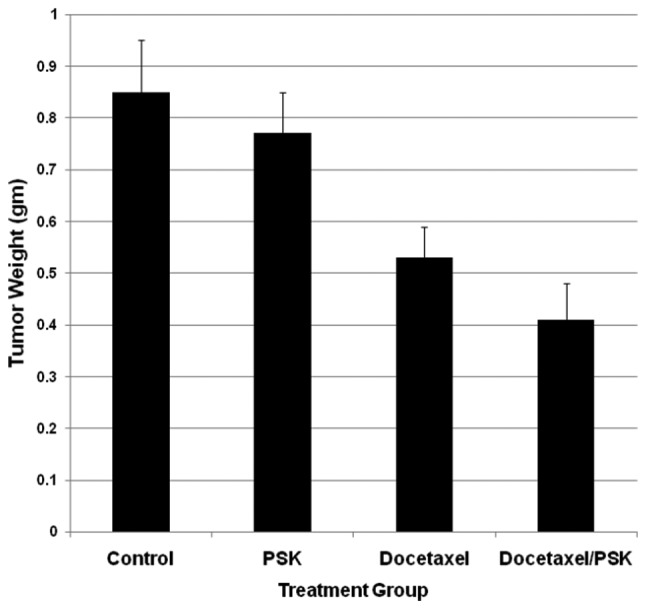
Oral administration of PSK plus docetaxel induces tumor regression. C57BL/6 mice with established tumors (50 mg) were treated with oral saline control daily, oral PSK (300 mg/kg) daily, i.p. injection of docetaxel (2x weekly) or a combination of PSK and docetaxel for a period of 10–12 days. Mean tumor weight ± standard error measurement (mean ± SEM) are shown for each treatment group of mice (n=7/group). PSK combined with docetaxel significantly suppressed tumor growth as compared to saline control, PSK or docetaxel treatments alone (P<0.05).

To determine if PSK enhances tumor suppression induced by docetaxel, groups of mice (n=10) bearing established TRAMP-C2 tumors were treated with saline control; subtherapeutic doses of docetaxel (5 mg/kg i.p. twice weekly); PSK (300 mg/kg orally by gavage once daily) or a combination of the docetaxel plus PSK (following the same dosing regimens as single agent treatments). A subtherapeutic dose of docetaxel was used to allow observation of enhancing effects of the combination treatment, consistent with previous studies that showed direct antitumor effects of adding intraperitoneally administered PSK to docetaxel treatment in mouse models of gastric cancer (26,27). Doses of up to 1,000 mg/kg PSK have been used in other studies with no reported toxicities (29). The combination of docetaxel plus PSK resulted in significantly smaller tumors, relative to saline control, docetaxel or PSK alone (Fig. 1) (p<0.05), though PSK-treated tumors were not significantly different than control.

**Figure 2:**
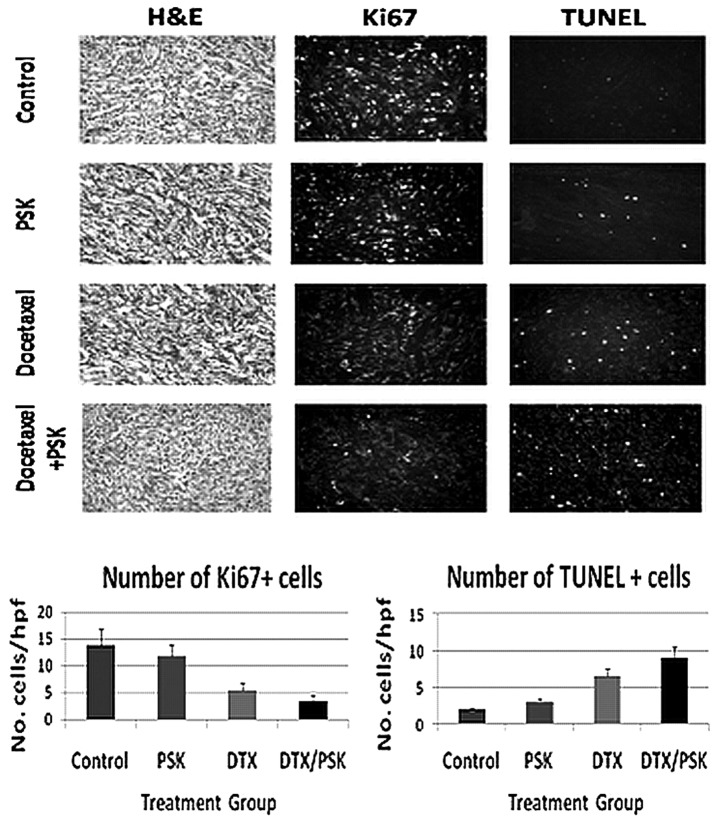
Ki67 and TUNEL expression in tumors treated with docetaxel and PSK. Mice with established prostate tumors were treated with oral saline daily, oral PSK (300 mg/kg) daily, i.p. docetaxel injection (2x weekly) or a combination of PSK and docetaxel for a period of 10–12 days. Tumors were stained for the Ki67 proliferation marker and TUNEL expression. Shown are representative staining results and summary graphs showing the number of tumor cells positive for each marker (mean ± SEM) viewed at magnification ×40 for a total of 10 views in each of 6–8 tumors (hpf, per high powered field; H&E, hematoxylin and eosin staining). Combining PSK with docetaxel induced a reduction in proliferating Ki67^+^ cells compared to docetaxel alone (p<0.05), and significantly enhanced the apoptotic, TUNEL^+^ cells compared to PSK alone (p<0.01) or docetaxel alone (p<0.05).

In order to assess potential mechanisms by which PSK enhances the effect of docetaxel induced tumor regression, tumors from treated mice were stained for Ki67 (proliferation) and annexin V (apoptosis). Combining PSK with docetaxel resulted in a reduction of Ki67 positive cells compared to docetaxel alone (p<0.05), and induced a 3-fold increase in the number of apoptotic, TUNEL positive cells compared to therapy with PSK (p<0.01), and a 1.5-fold increase compared to docetaxel alone (p<0.05) (Fig. 2).

### Combined docetaxel and PSK treatment does not cause adverse effects

**Figure 3:**
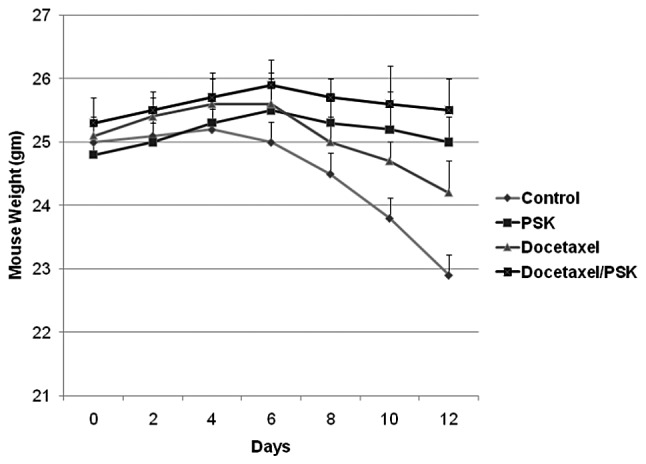
Change in murine weights with combination therapy. Mice used in the same experiment as in Fig. 2 were weighed every other day for 12 days. Data are shown as mouse weight (mean ± sem) of each group (n=7 per group). Saline control or docetaxel treatments alone led to significant loss in murine weight by day 12, while PSK alone or PSK combined with docetaxel treatment maintained murine weight over this time period.

Given the apoptosis-inducing effects that we observed, we investigated weight, hepatic and renal function in mice treated with PSK or docetaxel alone or in combination. No significant differences in liver and kidney function were observed between the treatment groups (Table I). Interestingly, weight was actually maintained with addition of PSK to the docetaxel regimen as compared to either the control or docetaxel groups (Fig. 3), suggesting a protective effect of the combined treatment against tumor-induced weight loss. Thus, no significant toxicity was observed with the PSK and docetaxel combination therapy. We next asked whether the combined therapy could allay observed immunosuppressive effects of docetaxel.

### PSK reduces docetaxel-induced immune suppression

**Figure 4:**
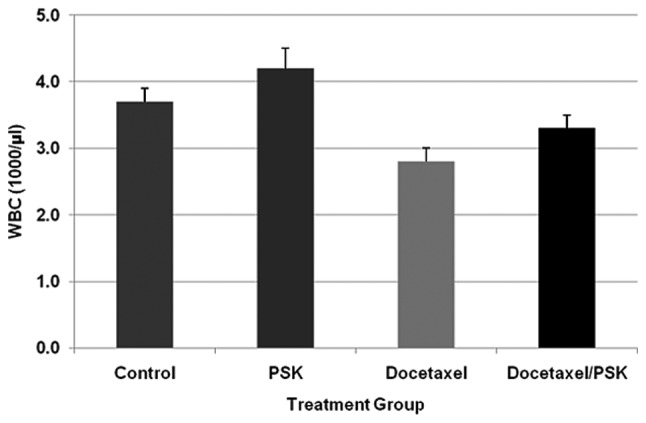
WBC in tumor bearing mice treated with PSK and docetaxel. Groups of mice (n=7) bearing established TRAMP-C2 tumors (12 days) were treated with either oral saline, subtherapeutic dose of docetaxel (5 mg/kg twice weekly), oral gavage of PSK (300 mg/kg), or a combination of docetaxel and PSK. WBC were assessed at the end of the experiment (mean ± SEM). Docetaxel alone induced a 25% reduction in WBC. Addition of PSK decreased the docetaxel-suppressive effect by ~50%, a difference that was statistically significant compared to docetaxel alone (p=0.03).

To determine the impact of PSK on docetaxel-induced immunosuppression, WBC counts were determined. Docetaxel alone induced a 25% reduction in WBC, and addition of PSK decreased this suppressive effect by ~50%. The difference in WBC level observed in response to PSK + docetaxel treatment compared to docetaxel alone was statistically significant (p=0.03) (Fig. 4). Whether this effect is protective (inhibits chemotherapy-induced lymphatic suppression) or restorative (increases WBC levels after therapy) remains to be determined.

### Combining PSK with docetaxel enhances tumor infiltrating lymphocyte (TIL) numbers

**Figure 5:**
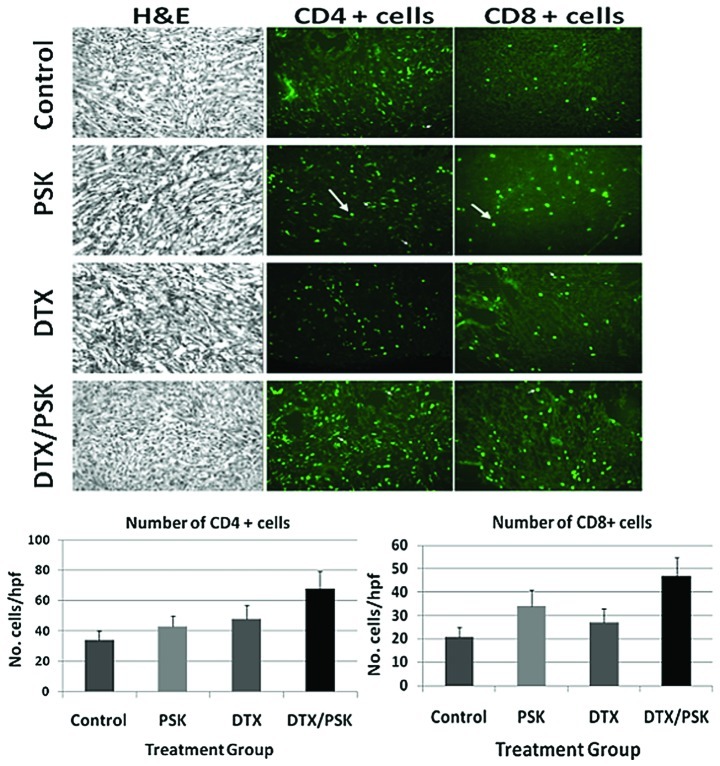
PSK combined with docetaxel enhances tumor infiltrating T cells. Mice bearing orthotopic TRAMP-C2 tumors were treated with either: 1) saline; 2) PSK (300 mg/kg daily; 3) docetaxel (5 mg/kg); or 4) PSK and docetaxel. Mice were sacrificed on day 12 after treatment and tumors were harvested. (A) Fluorescent immunohistochemistry was performed for CD4^+^ and CD8^+^ T cells (examples of positive cells indicated by arrows). Populations of both CD4^+^ (B) and CD8^+^ (C) T cell subsets were significantly higher in mice treated with docetaxel and PSK compared to PSK or docetaxel alone (p<0.05).

Since PSK enhanced antitumor responses in a Her2/neu breast cancer mouse model (17), we investigated whether combining PSK with docetaxel could enhance measures of antitumor immunity in the TRAMP-C2 model. First we asked whether numbers of TILs changed in response to the individual and combined treatments. Tumor sections were stained for expression of tumor infiltrating CD4^+^ and CD8^+^ T cells. The PSK and docetaxel treatments alone resulted in higher numbers of TILs than in control mice, and the addition of PSK to docetaxel increased both tumor infiltrating CD4^+^ T cells compared to PSK or docetaxel alone (p<0.05) and CD8^+^ T cell numbers compared to docetaxel alone (p<0.05) (Fig. 5), suggesting an enhancing effect of the combined treatment on tumor infiltrating T cells.

### Effects of PSK and docetaxel treatments on mRNA expression of cytokine and cytolytic genes and FoxP3

**Figure 6:**
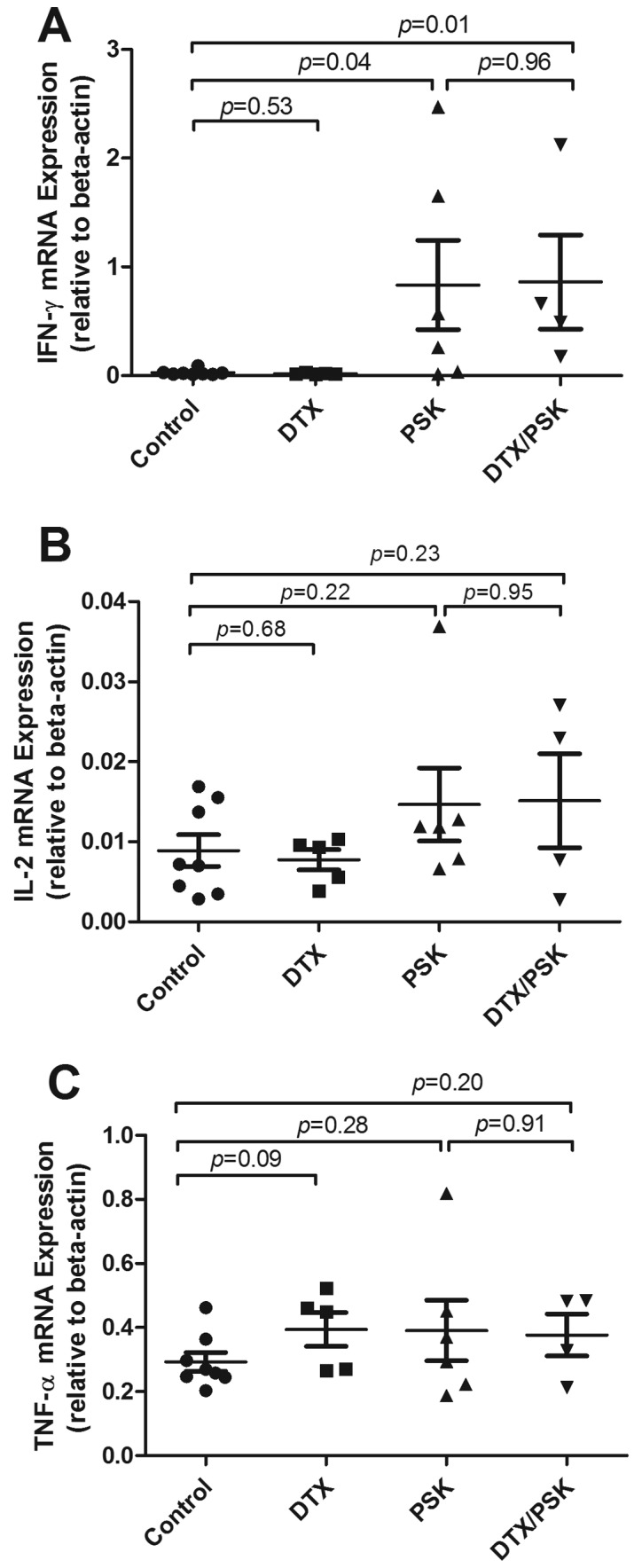
Effects of PSK and docetaxel treatments on mRNA expression of markers of antitumor immune response. Groups of mice (n=8 control; n=5 docetaxel; n= 6 PSK; n=4 PSK + docetaxel) bearing established TRAMP-C2 tumors (12 days) were treated with either oral saline, subtherapeutic dose of docetaxel (5 mg/kg twice weekly), oral gavage of PSK (300 mg/kg), or a combination of docetaxel and PSK. RNA was extracted from isolated tumors and real-time RT-PCR performed using primers and probes for several different cytokines and antitumor immune response markers. Shown are the effects of the different treatment groups on mRNA expression of IFN-γ (A), IL-2 (B), and TNF-α (C). The values shown are relative cytokine mRNA per 1,000 copies of β-actin. Mice treated with PSK, either alone (p=0.04) or with docetaxel (p=0.01) significantly induced IFN-γ mRNA expression in TRAMP-C2 tumors compared to saline control.

Given the observation of PSK and docetaxel-induced TIL enhancement, we further analyzed whether any changes in IFN-γ, IL-2, TNF-α, TGF-β, perforin, granzyme B and FoxP3 mRNA expression in the tumor micro-environment could be observed in response to the treatments. TRAMP-C2 prostate tumors from the 4 different treatment groups of mice were assessed using RT-PCR for the presence of mRNA of cytokines and other antitumor immune response markers, as well as for the T-regulatory cell marker, FoxP3. Docetaxel treatment alone did not induce IFN-γ. Significant induction of interferon gamma (IFN-γ) mRNA expression in TRAMP-C2 tumors occurred in mice treated with PSK, either alone (p=0.04) or with docetaxel (p=0.01) compared to saline control (Fig. 6A). No significant differences between the treatment groups were observed for the other markers examined. However, mean IL-2 mRNA expression in response to combined PSK and docetaxel treatment was higher than docetaxel treatment alone (Fig. 6B). Mean levels of tumor necrosis factor-alpha (TNF-α) mRNA in response to docetaxel or PSK treatment alone or in combination trended toward an increase compared to control (p=0.09) (Fig. 6C). No significant differences in perforin, granzyme B, TGF-β or FoxP3 mRNA expression were observed between treatment groups (data not shown).

### PSK and docetaxel treatments do not affect splenic immune cell population percentages or apoptosis of splenic T cells

Since PSK enhanced docetaxel-induced apoptosis of tumor cells, we next investigated whether this effect was specific to tumor cells or if PSK treatment also modulates apoptosis induction of splenic immune cell sub-sets. Immunophenotyping was used to analyze distribution of splenic lymphocyte sub-populations alone and with annexin V, a marker of apoptosis. No significant differences in distribution of splenic T cells, NK cells, B cells or dendritic cells (DC) between treatment groups were observed (data not shown). Further, levels of apoptotic cells among the splenic sub-populations were not significantly different in response to treatments compared to control. Thus, the PSK and docetaxel treatments alone or combined do not appear to induce splenic cell apoptosis.

We attempted to take a similar approach to assess the effect of PSK and docetaxel treatments on apoptosis in tumor-infiltrating T cells. We were unable to disassociate the tumors specimens sufficiently to create single cell suspensions needed for flow cytometry. As an alternative strategy, 8 frozen tumor sections from mice in each treatment group were processed for dual immunohistochemistry, staining for the caspase-3 apoptosis marker and CD4 or CD8 T cell markers. Relatively little apoptosis among CD4 or CD8 cells was observed, regardless of type of treatment (data not shown).

### PSK combined with docetaxel enhances splenic NK cell cytolytic activity

**Figure 7:**
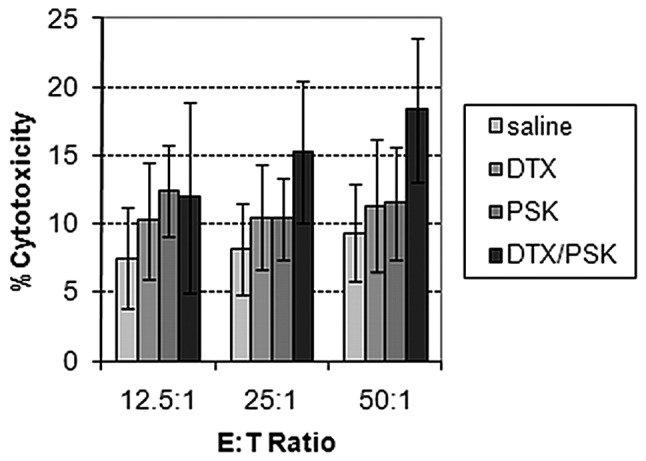
PSK in combination with docetaxel enhances NK cell activity of splenocytes from TRAMP-C2 tumor bearing mice. Splenocytes from mice treated with saline control or PSK and docetaxel alone and in combination were isolated and cultured *in vitro* with YAC-1 murine tumor target cells and assessed for NK cell killing of YAC-1 cells. Splenic cells from animals treated with PSK and docetaxel combination therapy had the highest level of NK cell activity at all effector:target ratios. NK cell activity induced by DTX/PSK was significantly enhanced at the 50:1 E:T ratio (p=0.045 using a linear mixed model to analyze data from 17 mice).

Spleens were collected and preserved from additional mice from each treatment group to assess the influence of PSK alone and combined with docetaxel on NK cell activity against YAC-1 tumor target cells. PSK and docetaxel combination therapy was found to have the greatest NK cell-inducing activity (Fig. 7). The splenic NK cell activity in mice treated with the combination of PSK and docetaxel was significantly higher compared to control mice at the 50:1 E:T ratio (p=0.045).

## Discussion

The TRAMP C2 tumor-bearing mouse model has frequently been used as a model to develop novel prostate cancer cytostatic and immunotherapy approaches (30–33). In this study we used this model to assess the modulatory effects of combination therapy with PSK and docetaxel treatment. Our results demonstrate that the addition of PSK to docetaxel has several beneficial effects. Addition of PSK to standard chemotherapy (docetaxel) enhanced tumor regression, prostate carcinoma apoptosis and proliferation compared to docetaxel alone. The combination of PSK and docetaxel was also immunoprotective, in that animals treated with the combination had less of a decrease in WBC compared to those treated with docetaxel alone. This was accompanied by an increase in the numbers of CD4^+^ and CD8^+^ T cells within the tumor. PSK treatment alone or with docetaxel significantly enhanced IFN-γ mRNA expression in tumors compared to the near absence in tumors from control and docetaxel groups. PSK combined with docetaxel also enhanced splenic NK cell cytolytic activity against tumor target cells.

These results are consistent with published studies showing the capacity of PSK to enhance gene expression of cytokines (34) and to induce TLR2-dependent activation pathways driving antitumor immune responses (17). PSP, a similar proteopolysaccharide extract isolated from *T. versicolor* has been reported to induce IFN-γ production and enhance NK cell tumoricidal activity in experimental animal cancer models (35). IFN-γ enhancement by polysaccharide-containing extracts from *Ganoderma lucidum* (Reishi) and *Grifola frondosa* (Maitake) mushroom species has also been reported (36–38). In this study we show that when given in conjunction with traditional, front-line docetaxel therapy, PSK administration was associated with smaller tumor size, more adenocarcinoma apoptosis and higher amounts of TILs. Collectively, these results support the clinical testing of the combination of PSK with taxol based therapy, such as docetaxol.

Studies in other cancer models have reported PSK-enhancing effects of docetaxel-induced tumor cell apoptosis (26,27). These studies of apoptosis induction mechanisms suggest that this apoptosis-enhancing effect may be a result of PSK inhibiting NFκB-induced expression of survivin, leading to a higher level of docetaxel-induced caspase-3 activation in tumor cells (25,26). These studies did not assess the effects of PSK addition to docetaxel on apoptosis induction in immune cells, so whether these effects are tumor-specific is unknown. In this study, we were unable to detect significant changes in splenic immune cell sub-population percentages of total mononuclear cells (data not shown). We therefore conclude that this combination appears to not affect normal lymphocytes. Moreover, the T cell apoptosis within tumor samples was low from all treatment groups. Thus, the ability of PSK to protect against apoptosis of tumor infiltrating lymphocytes was unclear. Docetaxel has been reported to induce apoptosis in mitogen-activated human peripheral blood mononuclear cells (PBMC) *ex vivo* without affecting Th1 cytokine production (39). Further studies examining this question in more depth are needed to determine whether the apoptosis-inducing effects of combined PSK and docetaxel treatment are tumor-specific.

Immunomodulatory effects of docetaxel have also been reported. Docetaxel is known to induce myelosuppression and reduce WBC counts in a time and dose-dependent fashion (7,8,40) and data from this study suggest that PSK may mitigate these effects while enhancing antitumor responses. With respect to direct immunological effects, docetaxel treatment in mice bearing syngeneic transplanted tumors induced a significant increase in T cell and NK cell infiltration into tumors (41). In this study, docetaxel induced an increase in both CD4^+^ and CD8^+^ TILs over saline treatment, an effect that was enhanced by the addition of PSK. Markasz *et al* reported that docetaxel at therapeutic concentrations effectively inhibited NK cell-mediated killing without affecting NK cell viability (42). We did not observe inhibition of NK cell lytic activity against tumor cells in response to docetaxel treatment alone in the TRAMP-C2 tumor model. However, the ability of PSK to enhance NK cell activity raises the possibility that PSK administration may counter docetaxel-induced NK cell inhibition.

The dose of PSK (300 mg/kg) used in combination with docetaxel in these murine experiments is somewhat higher than has been used in human clinical trials (~75 mg/kg) (43–45). At these higher doses, we observed no toxicities of daily oral PSK administration. Other investigators have tested oral administration of up to 1,000 mg/kg PSK in a murine lung cancer model and found fewer treatment-related deaths in PSK-treated animals (29). Furthermore, higher doses of *Trametes versicolor* preparations (up to 9 gm daily) have been well tolerated in a human trial in breast cancer patients (Standish, unpublished data). Collectively, the results of this study in a TRAMP-C2 murine model of human prostate cancer suggest that the combination regimen of PSK and docetaxel warrants further study in humans to determine if this combination therapy is of clinical benefit in the treatment of advanced prostate cancer. A phase Ib dose escalation clinical trial to determine the maximum tolerated dose of PSK in combination with docetaxel is underway.

## Figures and Tables

**Table I:** Analysis of plasma parameters of liver and renal function in treated TRAMP-C2 mice.

Treatment	ALT (U/l)	AST (U/l)	Cr (mg/dl)	BUN (mg/dl)	Albumin (g/dl)	Total protein (g/dl)
Control	40±12	150±88	0.22±0.10	22.8±7.2	1.83±0.2	4.26±0.4
DTX	36±11	112±32	0.20±0.10	20.3±1.2	1.95±0.3	4.40±0.5
PSK	45±10	178±188	0.19±0.15	17.0±2.0	2.00±0.2	4.50±0.4
PSK + DTX	43±12	143±56	0.20±0.10	19.0±2.5	1.92±0.2	4.33±0.4

N=4 mice; ALT, alanine transaminase; AST, aspartate aminotransferase; Cr, creatinine; BUN, blood urea nitrogen.

## Abbreviations

ALT: alanine transaminase
AST: aspartate amino-transferase
BUN: blood urea nitrogen
CDNA: complementary deoxyribonucleic acid
Cr: creatinine
CRPC: castration-resistant prostate cancer
IFN-γ: interferon-gamma
IgG: immunoglobulin G
IL-2: interleukin-2
i.p.: intraperitoneal
mRNA: messenger ribonucleic acid
NK: natural killer
PBMC: peripheral blood mononuclear cells
PCR: polymerization chain reaction
PSK: polysaccharide-K
TGF-β: transforming growth factor beta
TIL: tumor infiltrating lymphocytes
TLR: toll-like receptor
TNF-α: tumor necrosis factor alpha
TRAMP: transgenic adenocarcinoma of the mouse prostate
WBC: white blood cell
